# Correlation analysis of cytokine levels and peripheral blood T lymphocyte subsets in systemic vascular inflammatory disease

**DOI:** 10.5937/jomb0-61241

**Published:** 2026-05-22

**Authors:** Zhonghan Lin, Xuanyang Dong, Junye Chen, Biao Li

**Affiliations:** 1 Nanjing Medical University, Clinical College of Nanjing Medical University, Nanjing Drum Tower Hospital, Nanjing, Jiangsu, China; 2 Sun Yat-sen University, Laboratory Department, First Affiliated Hospital, No. 58, Guangzhou, China; 3 The Affiliated Suzhou Hospital of Nanjing Medical University, Suzhou Municipal Hospital, Department of Cardiology, Suzhou, China

**Keywords:** systemic vascular inflammatory disease, clinical features, T lymphocyte subsets, cytokine levels, sistemsko vaskularno zapaljensko oboljenje, klini~ke karakteristike, subpopulacije T limfocita, nivoi citokina

## Abstract

**Background:**

To measure the levels of T-cell subsets andcytokines in the peripheral blood of patients with systemicvascular inflammatory disease and to analyse the influenceof different clinical characteristics of systemic vascularinflammatory disease patients on the levels of T-cell subsetsand cytokines in the peripheral blood.

**Methods:**

The case group included 80 patients with systemicvascular inflammatory disease who were diagnosed and trea -ted at our hospital between January 2021 and December2024. The control group consisted of 40 healthy volunteersrecruited from our hospital’s health assessment centre. Theproportions of helper T-cell (Th)1, Th17, and regulatory Tcell (Treg) subsets in peripheral blood were analysed usingflow cytometry. Serum levels of cytokine, including interleukin-2 (IL-2), interferon-g (IFN-<span style="color: rgb(32, 33, 34); font-family: sans-serif; font-size: 16px; background-color: rgb(248, 249, 250);">γ</span>), tumour necrosis factor-a(TNF-<span style="color: rgb(32, 33, 34); font-family: sans-serif; font-size: 16px; background-color: rgb(248, 249, 250);">α</span>), IL-4, IL-10, IL-17, and IL-6, were measured usingenzyme-linked immunosorbent assay (ELISA).

**Results:**

Compared with the control group, the expressionlevels of total T lymphoid subsets and CD8+ T and Th1 cellsin patients with systemic vascular inflammatory disease weresignificantly higher, and there was no statistically significantdifference in Th, Th17, or Treg cell expression between thegroups (P&gt;0.05). However, there were statistically significantdifferences between the two groups [68.75 (54.23, 80.32)]and [72.10 (62.23, 86.45)], [23.44 (12.89, 33.76)] and[31.46 (20.13, 45.26)], [10.67 (8.23, 12.35)] and [10.26,17.96 [25.39)], Z=-3.13, -4.54, -3.97 (all P values&lt;0.05).Compared with those in the control group, patients with systemic vascular inflammatory disease had significantly higherserum levels of IL-17, IL-6, TNF-<span style="color: rgb(32, 33, 34); font-family: sans-serif; font-size: 16px; background-color: rgb(248, 249, 250);">α</span>, IL-4, and IFN-<span style="color: rgb(32, 33, 34); font-family: sans-serif; font-size: 16px; background-color: rgb(248, 249, 250);">γ</span>, whereastheir IL-10 levels were significantly lower. The differenceswere statistically significant (Z=-4.32, -5.01, -8.18, -8.70,-3.48, and -8.30). The P values were all &lt;0.05. Comparedwith that in patients without related manifestations, IL-6expression was noticeably higher in systemic vascular inflammatory disease patients with arthritic symptoms and thosewith active systemic vascular inflammatory disease, and thedifference was statistically significant {pg/mL: [3.23 (2.98,5.35)] compared with [10.51 (6.72, 23.21)], [6.32 (4.79,8.93)] compared with [9.68 (6.97, 18.73)], Z=5.47, 8.76,P values &lt;0.05}. Compared with those in patients withoutrelevant clinical manifestations, TNF-<span style="color: rgb(32, 33, 34); font-family: sans-serif; font-size: 16px; background-color: rgb(248, 249, 250);">α</span> levels were significantly higher in patients with arthritis, ocular, or digestive tractmanifestations, and in patients with active systemic vascularinflammatory disease, whereas IFN-<span style="color: rgb(32, 33, 34); font-family: sans-serif; font-size: 16px; background-color: rgb(248, 249, 250);">γ</span> levels were substantiallyhigher in systemic vascular inflammatory disease patientswith digestive system lesions. The differences were statistically significant {pg/mL: [7.74 (6.89, 10.19)] compared with[39.84 (30.37, 50.61)], [6.12 (5.36, 9.89)] compared with[31.35 (15.71, 30.46)], [6.49 (4.78, 10.21)] comparedwith [19.89 (14.36, 36.21)]. [5.89 (4.61, 8.96)] than[27.91 (15.32, 37.81)], [6.89 (5.43, 14.86)] than [26.79(15.41, 31.56)], Z= 7.70, 6.84, 6.94, 9.47, 5.70, P values&lt;0.05}. However, there was no statistically significant difference in the expression levels of IL-4 and IL-17 between systemic vascular inflammatory disease patients with and without relevant clinical manifestations (P&gt;0.05).

**Conclusions:**

Patients with systemic vascular inflammatorydisease exhibit an imbalance of T lymphocyte subsets andcytokines, and disease activity and clinical classificationmay also involve such imbalances.

## Abstract (sr)

**Uvod:**

Ispitivan je nivo subpopulacija T ćelija i citokina uperifernoj krvi pacijenata sa sistemskim vaskularnim zapaljenskim oboljenjem, kao i analiza uticaja različitih kliničkihkarakteristika ovih pacijenata na nivoe T-ćelijskih subpopulacija i citokina u perifernoj krvi.

**Metode:**

U grupi ispitanika je bilo 80 pacijenata sa sistemskim vaskularnim zapaljenskim oboljenjem koji sudijagnostikovani i lečeni u našoj ustanovi u periodu odjanuara 2021. do decembra 2024. godine. Kontrolnugrupu činilo je 40 zdravih dobrovoljaca iz centra za sistematske preglede iste ustanove. Ekspresija subpopulacija T-pomoćničkih ćelija (Th1, Th17) i regulatornih T ćelija (Treg) u perifernoj cirkulaciji određivana jeprotočnom citometrijom. Nivoi citokina interleukina iinterferona-g (IFN-<span style="color: rgb(32, 33, 34); font-family: sans-serif; font-size: 16px; background-color: rgb(248, 249, 250);">γ</span>) u serumu mereni su metodom imunoenzimskog testa (ELISA), uključujući TNF-<span style="color: rgb(32, 33, 34); font-family: sans-serif; font-size: 16px; background-color: rgb(248, 249, 250);">α</span>, IL-4, IL10, IL-17 i IL-6.

**Rezultati:**

U poređenju sa kontrolnom grupom, pacijenti sasistemskim vaskularnim zapaljenskim oboljenjem imali suznačajno više nivoe ukupnih T limfocitnih subpopulacija,CD8+ T ćelija i Th1 ćelija, dok razlike u ekspresiji Th,Th17 i Treg ćelija nisu bile statistički značajne (P&gt;0,05).Međutim, postojale su statistički značajne razlike izmeđugrupa [68,75 (54,23–80,32)] i [72,10 (62,23–86,45)],[23,44 (12,89–33,76)] i [31,46 (20,13–45,26)], [10,67(8,23–12,35)] i [10,26–17,96 (25,39)], Z=-3,13, -4,54,-3,97 (sve P&lt;0,05). Pacijenti su imali značajno višeserumske nivoe IL-17, IL-6, TNF-<span style="color: rgb(32, 33, 34); font-family: sans-serif; font-size: 16px; background-color: rgb(248, 249, 250);">α</span>, IL-4 i IFN-<span style="color: rgb(32, 33, 34); font-family: sans-serif; font-size: 16px; background-color: rgb(248, 249, 250);">γ</span>, dok su nivoi IL-10 bili značajno niži (Z=-4,32, -5,01, -8,18, -8,70,-3,48, -8,30; svi P &lt;0,05). Kod pacijenata sa artritičnimsimptomima i aktivnim oblikom bolesti, ekspresija IL-6 bilaje značajno viša nego kod pacijenata bez tih manifestacija{pg/mL: [3,23 (2,98–5,35)] prema [10,51 (6,72–23,21)],[6,32 (4,79–8,93)] prema [9,68 (6,97–18,73)], Z=5,47,8,76, P&lt;0,05}. Nivoi TNF-<span style="color: rgb(32, 33, 34); font-family: sans-serif; font-size: 16px; background-color: rgb(248, 249, 250);">α</span> bili su značajno viši kod pacijenata sa artritičnim, očnim i gastrointestinalnim manifestacijama, kao i kod pacijenata sa aktivnom bole{}u,dok su nivoi IFN-<span style="color: rgb(32, 33, 34); font-family: sans-serif; font-size: 16px; background-color: rgb(248, 249, 250);">γ</span> bili povišeni kod pacijenata sa gastrointestinalnim lezijama (Z=7,70, 6,84, 6,94, 9,47, 5,70, sviP&lt;0,05). Nije bilo značajnih razlika u nivoima IL-4 i IL-17između pacijenata sa i bez odgovarajućih kliničkih manifestacija (P&gt;0,05).

**Zaključak:**

Pacijenti sa sistemskim vaskularnim zapaljenskim oboljenjem pokazuju disbalans subpopulacija T limfocita i citokina. Aktivnost bolesti i klinička klasifikacijamogu da budu povezane sa ovim imunološkim disbalansom.

## Introduction

Systemic vascular inflammatory disease is a chronic inflammatory disease with vasculitis as its pathological basis and involves multiple systems, with recurrent episodes. The damage mainly affects the oral cavity, external genitalia, and eyes and can also involve the visceral system. The different types of damage to the visceral system can be classified into vascular, neurological, gastrointestinal, etc. The aetiology and pathogenesis of this damage remain unclear. Early research revealed that a key factor in the pathophysiology of systemic vascular inflammatory disease is the helper T (Th)type 1 immunological response [1]. Research has revealed that Th17/regulatory T (regulatory T) cells are important for the onset and progression of systemic vascular inflammatory disease [1][2][3][4].

Systemic vascular inflammatory disease is a complex, chronic inflammatory condition that affects multiple systems and manifests as skin lesions, eye inflammation, external genital ulcers, and mouth ulcers [4]. Although its aetiology has not yet been fully clarified, many studies [5][6][7] have shown that immune system abnormalities, primarily imbalances in T lymphocytes, play important roles in its pathogenesis. Research on peripheral blood cytokines and T lymphocyte subsets has steadily increased in recent years, suggesting their possible roles in the aetiology and development of Behcet's disease [8]. Patients with systemic vascular inflammatory disease have aberrant peripheral blood T lymphocyte subset proportions, with Th1 and Th17 cells considerably increasing while regulatory T cells (Tregs) decline. The increase in these cytokines is closely related to disease activity. Domestic research also supports this view, showing that certain cytokines, such as IL-23 and IL-12, are significantly elevated in patients with systemic vascular inflammatory disease. In addition, studies on gene polymorphisms have shown that certain mutations in immune-regulatory genes may increase susceptibility to systemic vascular inflammatory disease [9][10][11]. However, the specific mechanisms of action of T lymphocyte subsets and cytokines in systemic vascular inflammatory disease remain incompletely understood, and differences among studies may be due to factors such as sample size, detection techniques, and variations in patient backgrounds [12]. In addition, major challenges in clinical practice remain in improving the diagnosis and treatment of systemic vascular inflammatory disease by regulating these immune indicators.

This study explored the influence of various clinical characteristics in patients with systemic vascular inflammatory disease on peripheral blood T-cell subsets and cytokine levels.

## Materials and methods

### Research subjects

Fifty-nine male and 21 female systemic vascular inflammatory disease patients were selected as research participants from 80 systemic vascular inflammatory disease patients hospitalised or observed in our hospital’s outpatient department between January 2021 and December 2024, and whose data were complete. The average age was 32.34+14.21 years, the median age of onset was 31 years, and the average disease duration was 3.76+11.62 years. Twenty-eight males and twelve females, with an average age of 34.25+15.32 years, were chosen as the control group from among the 40 healthy examinees whose sex and age were matched with the case group in our hospital’s health examination centre during the same period. This study has been approved by the Human Medical Research Ethics Committee (No. HKYS-2025-A0158).

### Test reagents and equipment

A FACSCalibur flow cytometer (systemic vascular inflammatory disease Biosciences, USA), a CO2 cell culture incubator (Thermo Fisher, USA), a centrifuge (Thermo Fisher, USA), an IFN-γ-FITC-antibody (Ebioscience, USA), an IL-17 PE, a CD3 Percp, a CD8-APC, the corresponding homolotype and corresponding membrane breakers, a PMA/TPA (PKC activator) (Biyutan, China), an ionomycin (ENZO, USA), and a Monensin (Ebioscience, USA) containing 10% fetal bovine serum 1640 medium for FCS and an enzyme-linked immunosorbent assay (ELISA) kit (Xinbosheng, Neobioscience, China) were used.

Flow cytometry detection Kit: systemic vascular inflammatory disease Biosciences, item number systemic vascular inflammatory disease 560882 (for labelling T lymphocyte subsets). Composition: Includes monoclonal antibodies such as CD3, CD4, CD8, CD25, CD45RO, etc., suitable for flow cytometry analysis. Cytokine Detection Kit: eBioscience, catalogue numbers BMS6002 (for ELISA detection of IL-6) and BMS6003 (for ELISA detection of TNF-α.). This kit is suitable for quantitative analysis of IL-6 and TNF-α. levels in peripheral blood. Lymphocyte isolation solution: Tsystemic vascular inflammatory disease Sciences, catalogue number: Tsystemic vascular inflammatory disease-105 (for isolation of peripheral blood mononuclear cells). This isolation solution can be used to efficiently isolate lymphocytes from peripheral blood. Flow cytometer: systemic vascular inflammatory disease Biosciences, model: systemic vascular inflammatory disease LSRFortessa, for analysing the fluorescence signals of T lymphocyte subsets. ELISA instrument: BioTek, model: BioTek ELx808, used for the detection and analysis of cytokine concentrations.

### Laboratory testing methods

This study aims to analyse the correlation between T lymphocyte subsets and cytokines in the peripheral blood of patients with systemic vascular inflammatory disease. The experiment was conducted using flow cytometry and enzyme-linked immunosorbent assay (ELISA) for detection.

### T lymphocyte subset detection

Flow cytometry uses fluorescently labelled antibodies to identify specific T cell surface markers and quantitatively analyses cell populations by laser excitation of fluorescence. Peripheral blood samples were collected from patients with systemic vascular inflammatory disease, and peripheral blood mononuclear cells were isolated using the lymphocyte isolation solution of Tsystemic vascular inflammatory disease Sciences (Catalogue number: Tsystemic vascular inflammatory disease-105). The cell suspension was mixed with the relevant fluorescently labelled antibody and incubated at room temperature in the dark for 30 minutes. Cell populations were detected by flow cytometry, and the proportion of T lymphocyte subsets was analysed.

### Cytokine detection

Enzyme-linked immunosorbent assay (ELISA) is based on antigen-antibody reactions. It uses enzyme-labelled secondary antibodies for signal amplification and quantitatively determines cytokine concentrations by measuring substrate colouration. Add the peripheral plasma sample to the Wells of the ELISA plate. Add the enzyme-labelled secondary antibody, incubate at room temperature, then wash. Add the substrate solution, incubate, and then measure the absorbance using an ELISA instrument to calculate the cytokine concentration.

### Detection methods

Peripheral venous blood was collected from systemic vascular inflammatory disease patients on an empty stomach in the morning (2 mL for heparin anticoagulant tubes and 2 mL for procoagulant tubes). Heparin-treated cells were subjected to flow cytometry within 4 hours, and Th1 (CD3+CD4+ IFN-γ+), Th17 (CD3+CD4+IL17+), and Treg (CD4+CD25+Foxp3+) cells were detected within 10 hours. One of the procoagulant blood tubes was used to separate the serum, which was aliquoted and frozen at -80°C for future use. The levels of the cytokines IFN-γ, IL-4, IL-10, IL-17, IL-6 and TNF-α were determined via ELISA. Wuhan Antujie Biotechnology Co., Ltd, carried out detection.

### Statistical analysis

SPSS 21.0 software was used to perform the statistical analysis. When the distribution was nonnormal and the variance was homogeneous, the median and interquartile range (M(Q1, Q3)) were used to express the data. For group comparisons, the Wilcoxon rank-sum test was used.

## Results

### General clinical data of systemic vascular inflammatory disease patients

All 80 patients with systemic vascular inflammatory disease had recurrent oral ulcers (100%). Fifty-five patients had genital ulcers (68.75%). Fifty-one patients (63.75%) had skin lesions, presenting as erythema nodosum and pseudofolliculitis. 13 patients (16.25%) had positive acupuncture reactions. There were 27 cases (33.75%) of ophthalmia, all of which were uveitis. There were 33 cases (41.25%) with digestive system lesions, including multiple ulcers in the gastrointestinal tract. There were 25 cases (31.25%) of circulatory system lesions, 19 cases (23.75%) of neurological lesions, and arthritis/arthralgia in 39 cases (48.75%). Among them, 48 patients were in the active stage of the disease, and 32 patients were in the stable stage when the samples were collected.

### Comparison of lymphocyte subsets between systemic vascular inflammatory disease patients and controls

**Figure 1:**
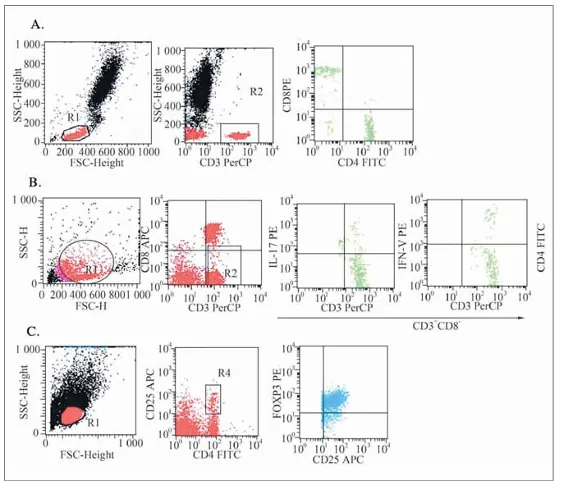
Flow cytometry detection of lymphocyte subsets in BD patients.<br>A. Flow cytometry of T lymphocyte subsets; B. Flow cytometry of Th17 and Th1 cells; C. Flow cytometry of Treg cells.

The total number of T lymphoid subsets and the percentages of CD8+ T and Th1 cells in systemic vascular inflammatory disease patients were significantly greater (P<0.05), whereas there were no statistically significant differences in the numbers of Th, Th17 or Treg cells between the groups (P>0.05) (Table 1, Figure 1).

**Table 1:** Comparison of lymphocyte subsets between systemic vascular inflammatory disease patients and the control group.

Project	Systemic vascular inflammatory <br>disease group (80 cases)	Control group <br>(40 cases)	Z value	P value
T (CD3+) (%)	72.10 (62.23, 86.45)	68.75 (54.23, 80.32)	-3.13	<0.05
Th (CD3+CD4+) (%)	35.81 (23.63, 47.82)	34.56 (20.37, 45.69)	1.54	>0.05
Ts (CD3+CD8+) (%)	31.46 (20.13, 45.26)	23.44 (12.89, 33.76)	-4.54	<0.05
Th1 (CD3+CD4+IFN-g+) (%)	17.96 (10.26, 25.39)	10.67 (8.23, 12.35)	-3.97	<0.05
Th17 (CD3+CD4+IL17+) (%)	5.88 (3.34, 12,21)	3.81 (2.83, 4.67)	-1.91	>0.05
Treg (CD4+CD25+Foxp3+) (%)	0.21 (0.04, 2.21)	0.08(0.02,1.56)	1.02	>0.05

### Comparison of cytokine expression levels between systemic vascular inflammatory disease patients and the control group

Serum IL-17, IL-6, TNF-α, IL-4, and IFN-γ expression levels were considerably higher in systemic vascular inflammatory disease patients than in control patients. In contrast, the amount of IL-10 was considerably reduced. Table 2 shows that the differences were statistically significant (P<0.05).

**Table 2:** Comparison of cytokine expression levels between systemic vascular inflammatory disease patients and the control group.

Cytokine	Systemic vascular inflammatory <br>disease group (80 cases)	Control group (40 cases)	Z value	P value
IFN-γ (pg/mL)	8.80 (4.30, 20.45)	4.78 (3.55, 7.50)	-3.48	<0.05
TNF-α (pg/mL)	12.13 (10.60, 17.24)	4.12 (1.95, 6.60)	-8.18	<0.001
IL-6 (pg/mL)	4.74 (3.59, 12.59)	3.20 (1.86, 4.41)	-5.01	<0.05
IL4 (pg/mL)	4.28 (3.50, 5.22)	1.07 (1.03, 1.12)	-8.70	<0.001
IL-10 (pg/mL)	0.55 (0.42, 0.76)	1.63 (1.33, 2.46)	-8.30	<0.001
IL-17 (pg/mL)	20.11 (10.31, 45.05)	8.59 (5.82, 13.38)	-4.32	<0.05

### Comparison of the expression levels of IL-6 and IL-10 in systemic vascular inflammatory disease patients with different clinical manifestations

Compared with that in systemic vascular inflammatory disease patients without arthritis manifestations, the serum IL-6 level in patients with arthritis manifestations was significantly higher. Compared with that in patients with stable systemic vascular inflammatory disease, the expression level of IL-6 in patients with active systemic vascular inflammatory disease was significantly greater (P<0.05) (Table 3). However, there was no statistically significant difference in IL-10 levels between patients with systemic vascular inflammatory disease without relevant clinical manifestations and those with relevant clinical manifestations (P>0.05).

**Table 3:** Comparison of L-6 expression levels in systemic vascular inflammatory disease patients with different clinical manifestations.

Clinical features	Number <br>of cases	IL6 (pg/mL)		Z value	P value
		Have relevant <br>manifestations	No relevant <br>manifestations		
Oral ulcer	80	4.74 (3.59, 12.59)	-	-	-
Vulvar ulcer	55	3.92 (3.13, 7.89)	5.63 (4.78, 13.63)	1.18	>0.05
Skin manifestations	51	4.21 (3.67, 9.06)	6.93 (5.47, 15.04)	1.49	>0.05
Arthritis	39	10.51 (6.72, 23.21)	3.23 (2.98, 5.35)	5.47	<0.05
Ophthalmia	27	4.32 (3.91, 9.28)	7.37 (5.52, 16.24)	1.92	>0.05
Lesions of the digestive system	33	5.69 (4.78, 10.53)	4.28 (3.68, 7.32)	1.62	>0.05
Circulatory system lesions	25	5.38 (4.69, 7.34)	4.78 (3.86, 7.39)	1.50	>0.05
Neurological disorders	19	5.24 (4.93, 7.21)	4.89 (3.95, 8.03)	0.97	>0.05
Disease activity	48	9.68 (6.97, 18.73)	6.32 (4.79, 8.93)	8.76	<0.05

### Comparison of TNF-α and IFN-γ expression levels in systemic vascular inflammatory disease patients with different clinical manifestations

Compared with those in patients without relevant clinical manifestations, TNF-α expression levels in systemic vascular inflammatory disease patients with arthritic, ocular, or digestive tract manifestations were significantly higher, whereas IFN-γ expression levels in systemic vascular inflammatory disease patients with digestive system lesions were substantially higher. The differences were statistically significant (Table 4 and Table 5). However, there was no statistically significant difference in the expression levels of IL-4 and IL-17 between systemic vascular inflammatory disease patients with and without relevant clinical manifestations (P>0.05).

**Table 4:** TNF-α levels in systemic vascular inflammatory disease patients with different clinical manifestations.

Clinical features	Number <br>of cases	TNF-α (pg/mL)		Z value	P value
		Have relevant <br>manifestations	No relevant <br>manifestations		
Oral ulcer	80	12.13 (10.60, 17.24)	-	-	-
Vulvar ulcer	55	16.63 (13.48, 20.78)	8.91 (7.63,12.39)	1.55	>0.05
Skin manifestations	51	17.68 (17.89, 23.65)	9.96 (8.89, 12.81)	1.48	>0.05
Arthritis	39	39.84 (30.37, 50.61)	7.74 (6.89, 10.19)	7.70	<0.05
Ophthalmia	27	31.35 (15.71,30.46)	6.12 (5.36, 9.89)	6.84	<0.05
Lesions of the digestive system	33	19.89 (14.36, 36.21)	6.49 (4.78, 10.21)	6.94	<0.05
Circulatory system lesions	25	16.67 (12.14, 20.21)	9.86 (7.67, 10.37)	1.76	>0.05
Neurological disorders	19	15.78 (10.63, 17.46)	10.56 (8,75, 11.34)	1.65	>0.05
Disease activity	48	27.91 (15.32, 37.81)	5.89 (4.61, 8.96)	9.47	<0.05

**Table 5:** Comparison of IFN-γ expression levels in systemic vascular inflammatory disease patients with different clinical manifestations.

Clinical features	Number <br>of cases	IFN-γ (pg/mL)		Z value	P value
		Have relevant <br>manifestations	No relevant <br>manifestations		
Oral ulcer	80	8.80 (4.30, 20.45)	-	-	-
Vulvar ulcer	55	9.64 (5.43, 15.89)	8.89 (5.14, 16.68)	1.82	>0.05
Skin manifestations	51	9.23 (4.65, 20.36)	8.95 (3.98, 18.23)	1.68	>0.05
Arthritis	39	10.98 (5.67, 21.86)	7.23 (4.32, 19.73)	1.91	>0.05
Ophthalmia	27	10.35 (4.67, 17.35)	7.37 (4.32, 19.01)	1.87	>0.05
Lesions of the digestive system	33	26.79 (15.41, 31.56)	6.89 (5.43, 14.86)	5.68	<0.05
Circulatory system lesions	25	9.01 (4.89, 21.87)	7.86 (3.67, 16.92)	1.76	>0.05
Neurological disorders	19	10.86 (5.03, 23.47)	8.92 (4.01, 17.65)	1.87	>0.05

## Discussion

Systemic vascular inflammatory disease is a chronic inflammatory illness that can affect multiple systems, including the gastrointestinal tract, blood vessels, and the central nervous system [13][14][15]. The pathogenesis of systemic vascular inflammatory disease is complex, and T lymphocytes and related cytokines play important roles in its pathogenesis [16]. Early studies revealed that the Th1 immune response is involved in the pathogenesis of systemic vascular inflammatory disease. Th1-related cytokines, such as IFN-γ and TNF-α, are significantly elevated in patients with systemic vascular inflammatory disease [17]. Research has shown that Th17 cell levels in patients with systemic vascular inflammatory disease are elevated considerably [18]. Compared with the normal control group and the stationary systemic vascular inflammatory disease group, patients with active systemic vascular inflammatory disease had higher levels of CD4+CD25+ Tregs and higher levels of Foxp3 mRNA expression [19][20][21]. Although the Th17 and Treg cell levels in the systemic vascular inflammatory disease patient group were higher than those in the normal control group, the difference was not statistically significant. One possible explanation is that the study included fewer patients.

A study [22] on IL-17, IL-10, IL-6, and IFN-γ in patients with systemic vascular inflammatory disease revealed that IL-17 and IL-6 were expressed at high levels, whereas IL-10 was expressed at low levels. Our experimental results revealed that the levels of IL-4, IL-17, IL-6, TNF-α and IFN-γ in the case group were significantly greater than those in the normal control group, whereas the level of IL-10 decreased significantly, which was basically consistent with reports in the abovementioned literature. 1L-6 is a multifunctional proinflammatory factor that regulates immune responses and is elevated in various immune and inflammatory diseases [23]. According to preliminary research, Tregs mediate their immunosuppressive activity throughout the pathophysiology of systemic vascular inflammatory disease by utilising immuno-suppressive substances and by affecting the secreted products of these cells (such as Th1 cells). Th17 cells (macrophages) have both negative and positive feedback effects. IL-4 is a cytokine related to Th2 cells [24][25][26][27]. Compared with the control group, IL-6 and IL-4 expression were significantly increased in patients with systemic vascular inflammatory disease, whereas IL-10 expression was significantly decreased. The expression level of IL-10 in patients with systemic vascular inflammatory disease was decreased considerably, suggesting that IL-10, a negative inflammatory factor, can inhibit the body’s immune response.

Currently, relatively few reports on lymphocyte subsets and cytokines in patients with systemic vascular inflammatory disease exist, and the results are not entirely consistent. The reason may be differences in the pathogenesis of systemic vascular inflammatory disease and the complex, diverse clinical phenotypes [28]. Our research revealed that serum IL-6 levels were significantly elevated in patients with systemic vascular inflammatory disease in the active stage and with arthritis manifestations. Tocilizumab (TCZ), a humanised IL-6 inhibitor, has achieved specific therapeutic effects. Studies [29][30][31] have shown that the serum level of IL-10 in patients with systemic vascular inflammatory disease is positively correlated with ocular manifestations, whereas the serum level of IFN-γ is negatively correlated with ocular manifestations [32]. The immune response in these patients is believed to be mainly mediated by Th1 cells rather than Th17 cells. The pathogenesis of gastrointestinal systemic vascular inflammatory disease may differ from that of other systemic vascular inflammatory diseases [33][34][35]. Another study [36] revealed that levels of IL-17 and IL-23, which are associated with Th17 cells, and of IL-35 and IL-10 were significantly increased in the intestinal systemic vascular inflammatory disease group. This study revealed that, compared with those in patients without digestive tract manifestations, the IFN-γ and IL-17 levels in systemic vascular inflammatory disease patients with digestive tract manifestations were slightly greater. The heterogeneity of the above studies may be due to differences in study populations, specimen collection methods (local tissue fluid vs. tissue or serum), and other factors.

## Conclusion

The clinical manifestations of systemic vascular inflammatory disease vary, and its pathogenesis is complex. Further in-depth research with larger sample sizes is still needed. Given the limited sample size in this study, it is necessary to increase the sample size further and improve monitoring methods in the future to provide a basis for understanding the pathogenesis and treatment of systemic vascular inflammatory disease.

## Dodatak

### Conflict of interest statement

All the authors declare that they have no conflict of interest in this work.
